# Reporting violations of European Charter of Patients’ Rights: analysis of patient complaints in Croatia

**DOI:** 10.1186/s12910-021-00714-3

**Published:** 2021-11-08

**Authors:** Jasna Karačić, Marin Viđak, Ana Marušić

**Affiliations:** 1 Cochrane Croatia, University of Split School of Medicine, Split, Croatia; 2 Department of Research in Biomedicine and Health, University of Split School of Medicine, Split, Croatia

**Keywords:** Right to healthcare, Informed consent, Quality of health care, Health ethics, Patient perspective

## Abstract

**Background:**

The European Charter of Patients' Rights (ECPR) presents basic patients' rights in health care. We analysed the characteristics of patients' complaints about their rights submitted through the official complaints system and to a non-governmental organization in Croatia.

**Methods:**

The official system for patients’complaints in Croatia does not have a common pathway but offers different modes for addressing patient complaints. In this cross-sectional study, we analysed the reports about patients’ complaints from the official regional committees sent to the Ministry of Health. We also analysed the complaints received by the Croatian Association for the Protection of Patient’s Rights (CAPR) and mapped them to the ECPR.

**Results:**

The aggregated official data from the Ministry of Health in 2017 and 2018 covered only 289 individual complaints from 10 out of 21 counties. Complaints were most frequently related to secondary and tertiary healthcare institutions and details were not provided. CAPR received a total of 440 letters, out of which 207 contained 301 complaints about violations of patients’ rights in 2017–2018. The most common complaint was the Right of Access to health care (35.3%) from the ECPR, followed by the Right to Information (29.9%) and the Right to Safety (21.7%). The fewest complaints were about the Right to Complain (1.9%), Right to Innovation (1.4%), Right to Compensation (1.4%), and Right to Preventive Measures (1.0%).

**Conclusions:**

Reporting and dealing with patients’ complaints about violations of their patients’ rights does not appear to be effective in a system with parallel but uncoordinated complaints pathways. Mapping patient's complaints to the ECPR is a useful tool to assess the perception of patients’ rights and to plan actions to improve the complaints system for effective health care.

## Background

The rights of patients in Europe have been defined by the European Charter of Patients’ Rights (ECPR), which was drafted in collaboration with 12 citizens’ organizations from different EU countries in 2002 [1]. The document lists 14 patients’ rights [2]: 1. Right to Preventive Measures; 2. Right of Access; 3. Right to Information; 4. Right to Consent; 5. Right to Free Choice; 6. Right to Privacy and Confidentiality; 7. Right to Respect of Patients’ Time; 8. Right to the Observance of Quality Standards; 9. Right to Safety; 10. Right to Innovation; 11. Right to Avoid Unnecessary Suffering and Pain; 12. Right to Personalized Treatment; 13. Right to Complain; and 14. Right to Compensation.

These rights aim to guarantee a “high level of human health protection”, as defined by Article 35 of the Charter of Fundamental Rights of the European Union [3] and assure high quality of services provided by the national health services in Europe. A recent analysis of the national legislature of the EU member states showed a varying degree of implementation of these 14 rights in national laws and regulations [4]. The rights to information, consent, care quality, and prevention are more often covered by existing national laws compared to the rights to avoid pain, the right to innovation, and the right to respect patients’ time [5].

The protection of patients’ rights in different countries varies based on differences in the laws, organization of the healthcare service as well as economic, social, cultural, religious, and moral values [6]. The extent of effective implementation of the ECPR also varies across the EU. In some countries there are not specific provisions, but the laws regarding informed consent, privacy, and access to the health record also apply to health care [7].

One way to study how the rights of the patients are addressed in the healthcare system is the analysis of patient complaints. Although they are often unstandardized and provide an emotional description of individual patient experience [8], they are a valuable source of information on safety and one of the indicators of the quality of care or potential problems [9]. When analysed at an accumulated level, they can point out problematic trends in the health system [10], and can be used to improve patient safety, quality of healthcare, ethical culture, and clinical care [11]. European countries have different approaches to solving patients’ complaints, either through ombudsmen, different institutional and hospital boards, and/or courts [12]. Previous research of the European Convention on Human Rights has emphasized that most of the EU member countries have laws on defining and implementing patients’ rights, except Austria, Bulgaria, Ireland, Italy, and Malta [13]. Finland, Netherlands, and Hungary belong to the pioneers of legally defining and implementing patients’ rights [14]. Public reporting about outcomes of complaints is practiced in Scandinavian countries (Denmark, Finland, Iceland, Norway) but not common in many other EU countries [15].

A study from Austria showed that the number of patient complaints grows at the rate of 15% annually, but legal lawsuits are rare and account for a small fraction of complaints [16]. In Germany, there were about 14 thousand allegations of malpractice in 2015, the majority regarding specialist medical care [17]. In a study conducted on patients in Slovenia, the most common causes of complaints were violations of legal rights, deterioration of health, unavailability, and loss of documentation [18].

The Republic of Croatia has a universal health care system regulated by the Healthcare Act [19]. The Health Insurance Act defines mandatory insurance based on the principles of solidarity and reciprocity, whose primary aim is the provision of accessible, high-quality services to patients, including patients’ rights and safety [20]. The healthcare system in Croatia is funded by the Croatian Health Insurance Fund, which is the only provider of mandatory health insurance. The Croatian Ministry of Health is responsible for health policy, including regulation and governance of healthcare [21]. Healthcare is provided by primary and secondary healthcare institutions. The management of secondary healthcare institutions (public hospitals and university hospital centres) is split between the central government and the counties as administrative governing units [22]. Primary health care is mostly managed privately and funded from the health insurance fund or organized under counties’ health centres.

As the newest EU member, Croatia has not been included in previous studies of patient rights implementation [19]. The rights of patients in Croatia are generally protected by the Healthcare Act from 1993, which was updated in 2020 [20]. In 2004, a special Act on the Protection of Patients’ Rights (PRPA) was adopted [23] as a result of the civil society initiative for legislative protection of patients’ rights, primarily the Croatian Association for the Protection of the Patients’ Rights (CAPR) [24]. CAPR is now considered a key non-government organization (NGO) dealing with patients’ rights in Croatia [25, 26]. It is active at both the national and European levels and is one of the most recognized Croatian NGOs in the field of patients’ rights protection [27].

Despite advocacy improvements and improved legal protection of patients in Croatia, there are still challenges regarding patients’ rights in practice. For example, informed consent forms used at the secondary and tertiary level of care have low readability and may not to be appropriate for the general population in Croatia [28]. Qualitative studies have identified several problems with patients’ rights, such as patient autonomy, lack of privacy, an authoritative approach from medical professionals, and protection of patient data [29]. To the best of our knowledge, there are no studies on patient complaints submitted to formal and informal bodies in Croatia. In this study, we analysed patient complaints about their rights in health care submitted to official bodies supervised by the Ministry of Health and to non-governmental CARP. Our aim was to gain a broader understanding of healthcare quality in the country, and to identify areas of special concern with regard to patients’ rights and their regional distribution.

## Methods

### Framework for the protection of patient rights in Croatia

Current Croatian legislation generally draws from the ECRP. The Patients’ Right Protection Act [26] includes 7 out of 14 rights outlined in the ERCP: Right of Access, Right to Information, Right to Consent, Right to Free Choice, Right to Privacy and Confidentiality, Right to Complain, and Right to Compensation. The Healthcare Act includes the Right to Preventive Measures [19], whereas the Right to Safety and Right to the Observance of Quality Standards are included in the Act on the Quality of Health and Social Care [30]. The Right to Innovation, Right to Avoid Unnecessary Suffering and Pain, and Right to Personalized Treatment are not covered by the current legislation in Croatia.

**Fig. 1:**
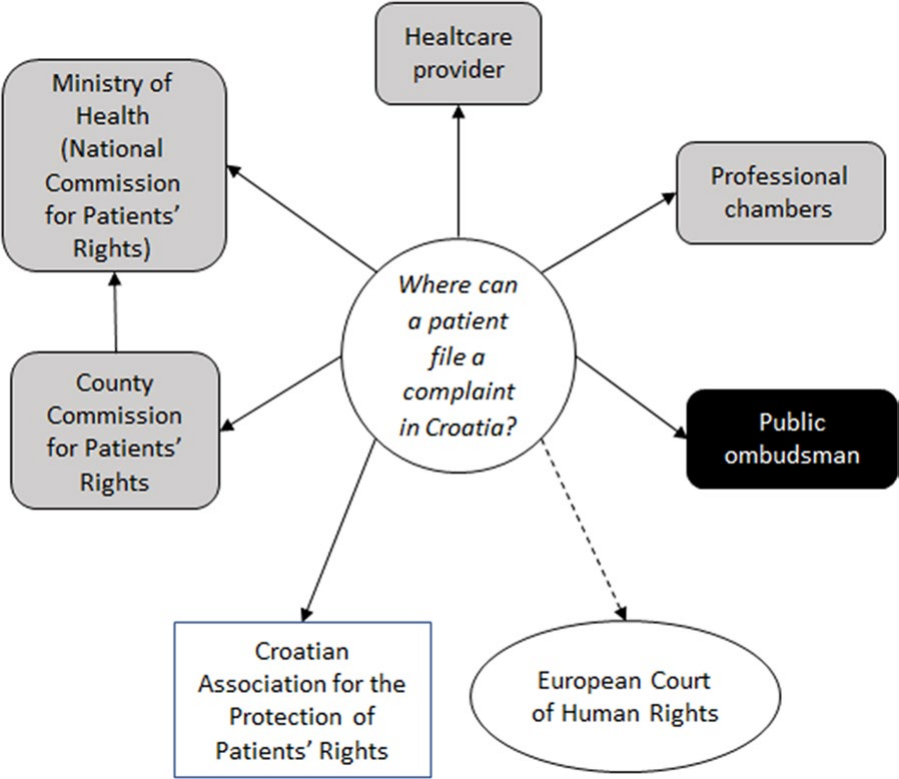
A flowchart of patient complaint process in Croatia, according to Healthcare Act, Protection of Patients’ Rights Act and the statute of the Croatian Association for the Protection of Patient Rights. Patients can file a complaint to one or more institutions independently. European Court of Human Rights is outside the national framework, where patients can directly submit a complaint against the Republic of Croatia

There is no single procedure or pathway for resolving patients’ complaints in Croatia. A patient can file a complaint independently and simultaneously to a number of institutions (Fig. 1), including the involved healthcare institution, directly to the Ministry of Health or through the regional committees for Protection of Patients’ Rights which report to the Ministry [31], as well as directly to the Public Ombudsman of the Republic of Croatia [32]. Healthcare providers are usually the first instance for patients’ complaints [33]. Each health institution is mandated to have a unit for insurance and improving the quality of healthcare and a Health Care Quality Commission [30]. The patients can also file complaints regarding the conduct of health professionals to their professional chambers, such as Croatian Medical Chamber or Croatian Chamber of Medical Nurses. These professional organizations monitor the protection of patients’ rights from the perspective of professional ethics and deontology [34].

Outside the institutional framework, the Croatian Association for the Protection of the Patients’ Rights (CAPR) is a non-profit, non-government organization that provides direct legal and expert advice to the patients [24]. CAPR is considered a key non-government organization dealing with patients’ rights in Croatia [27]. Finally, patients can also file complaints against the Republic of Croatia directly to the European Court of Human Rights if they think their rights, including patients’ rights, have been violated.

### Study design

We used a cross-sectional, retrospective descriptive study design to analyse two cohorts of patients’ complaints: (1) annual reports of the county committees sent to the Croatian Ministry of Health, and (2) patient complaints received via an official email to the non-governmental CAPR.

### Data sources

We analysed two sets of data: (1) reports submitted by 21 county committees to the Ministry of Health in 2017 and 2018, which we had obtained by a written request for public information [35] and (2) correspondence received via an official CAPR e-mail address in the same time period.

### Data analysis

#### Annual reports to the Ministry of Health

Only aggregated data were available, organized into categories by the type of health care profession/institution. Data are presented as absolute numbers of complaints per county committee and the type of institution included in the report.

#### Complaints to CAPR

For queries submitted to the CAPR, we analysed the texts of all reports to identify the rights addressed by the complainants. We used ECPR as a checklist to evaluate the content of complaints. Two authors (JK, MV) discussed each individual complaint and mapped (categorized) the complaints to one or more of 14 ECPR categories of patients’ rights [1]. In cases of disagreement, the third author (AM) was consulted, and agreement was reached by consensus. In cases where a complainant reported a violation of several ECPR rights, we counted those reports as a violation for each right separately (e.g., a complaint that addressed rights 2, 6 and 8, was accounted for three times, respectively). If the letter to the CAPR was not a complaint but a general question or request for information, we classified it as such. We also extracted the data on the age and gender of the person in the report, location of the health facility, level of care, and the type of health facility (public or private). The data are presented as absolute numbers of ECPR rights violations and overall percentages.

### Ethical considerations

The official written reports of the regional authorities were available in an aggregated form and were publicly available. Classification of the complaints to the CAPR was performed on a fully anonymised dataset provided by the CAPR officer responsible for patients’ complaints and was analysed by the two authors who are CAPR members (JK and MV). The third author, who served as an adjudicator for unclear cases (AM) did not have access to the database but was consulted by the two authors who provided necessary and anonymous information on the issue in question. Gender of the complainants could be inferred from anonymized texts; as Croatian language has a grammatical gender.

## Results

### Complaints to the Croatian Ministry of Health

**Fig. 2:**
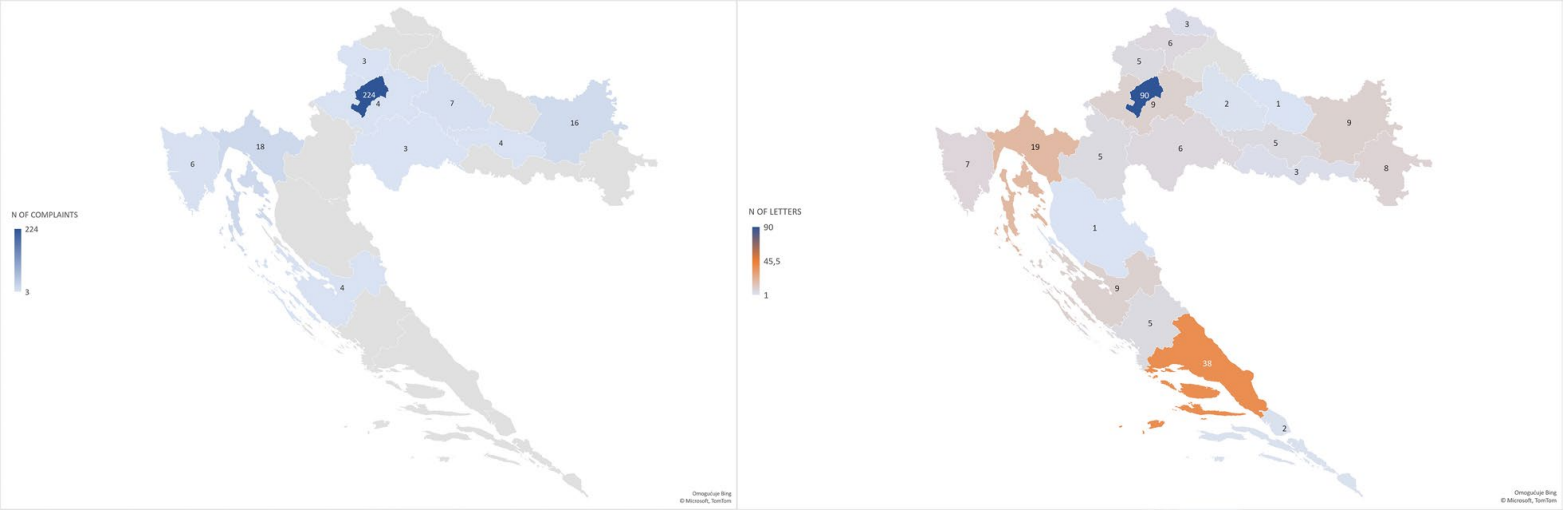
*Left:* Patient complaints to the regional committees in 20 administrative units (counties) and the City of Zagreb. For counties in grey, data from the Ministry of Health were not available; *Right:* Geographical origin of the letters to the Croatian Association for the Patients’ Rights (n = 237). Maps were generated using Excel for Microsoft 365

We received data on 289 individual complaints submitted from county committees to the Ministry of Health in 2017 and 2018. These complaints came from 10 regional committees. Data from the other 11 regional committees were not available. Out of 289 reports, 77.5% (n = 224) were from the City of Zagreb (Fig. 2: Left). Other counties had very low number of complaints, with the highest numbers for Primorje-Gorski Kotar County (6.2%) and Osijek-Baranja County (5.5%) (Fig. 2: Left).

These reports could not be mapped to the ECPR because the content of the complaint was not available. According to the official categorization of the Ministry, most frequent complaints were related to secondary and tertiary healthcare institutions (regional and university hospitals), followed by primary healthcare (family doctors and general practitioners), other (unspecified reports), and the Croatian Health Insurance Fund (Table 1**)**.

**Table 1:** Analysis of patients’ reports from the Ministry of Health, mapped according to institution and county included in the report (N = 289)

	Total number of reports	Zagreb (city)	Zagreb county	Osijek–Baraja	Bjelovar–Bilogorje	Krapina Zagorje	Istra	Primorje–Gorski kotar	Sisak–Moslavina	Zadar	Požega–Slavonia
Primary health care	59	43	1	3	3	2	1	4		2	
Secondary health care	77	56		5	1	1	2	8	2	1	1
Medical specialists	27	20	1		1		1	3			1
Dental medicine	9	6					1	1			1
Psychologists	3	3									
Nurses	20	19			1						
Croatian Health Insurance Fund	31	26		3				1		1	
Drugs’ committee	3			3							
Legal expertise	1								1		
Emergency medicine	3		1		1						1
Laboratory medicine	1		1								
Physical therapy	2						1	1			
Other	53	51		2							
Total	289	224	4	16	7	3	6	18	3	4	4

### Complaints to CAPR

CAPR received 440 e-mail letters from patients in 2017 and 2018. Out of those, 242 (55.0%) letters involved female and 151 (34.3%) male complainants, 6 (1.4%) letters involved patients of both genders, and in 41 (9.3%) letters the gender was not recorded or could not be inferred from the text of the complaint.

Details about the person or institution about which the complaint was made were not provided in 46.1% of the letters. The letters where this information was included (n = 237) came mostly from the capital of Zagreb (20.5%), followed by the Split-Dalmatia County (8.6%), which includes Split, the second-largest city in Croatia (Fig. 2: Right).

Most of the comments in patients’ letters (233, 52.9%) were general questions about the healthcare system in Croatia. In 207 letters that included complaints about the alleged violations of ECPR, we identified a total of 301 complaints that included violations of patients’ rights as outlined in the ECPR. The median number of complaints about patients’ rights violations was 1 (interquartile range = 1–2, range = 1–5).

Complaints about the Right of Access to health care were most common (n = 73, 35.3%), followed by complaints about the Right to Information (n = 62, 29.9%), the Right to Safety (n = 45, 21.7%), the Right to Respect of Patients’ Time (n = 38, 18.4%), and the Right to Avoid Unnecessary Suffering and Pain (n = 38, 18.4%). The Right to Complain, the Right to Innovation, the Right to Preventive Measures, the Right to Compensation and were least reported (fewer than 2% of the cases for each category; Table 2).

**Table 2:** Patients’ complaints to the Croatian Association for Patients’ Rights according to European Charter of Patients’ Rights (N = 207)

Charter right	N (%)*
Right of access	73 (35.3)
Right to information	62 (29.9)
Right to safety	45 (21.7)
Right to respect of patients’ time	38 (18.4)
Right to avoid unnecessary suffering and pain	38 (18.4)
Right to personalized treatment	8 (3.7)
Right to consent	7 (3.7)
Right to the observance of quality standards	7 (3.7)
Right to free choice	6 (2.9)
Right to privacy and confidentiality	5 (2.4)
Right to complain	4 (1.9)
Right to innovation	3 (1.4)
Right to compensation	3 (1.4)
Right to preventive measures	2 (1.0)

^*^Total number of charter rights violations is higher than a number of complaints analysed (N = 207) as some complaints included more than one charter right violations

The majority of complaints dealt with patients’ rights in secondary and tertiary healthcare institutions (n = 112, 54.1%), followed by primary healthcare providers (including general practitioners, family medicine doctors, primary care paediatricians, emergency medicine, and state-employed dental medicine doctors; n = 46, 22.2%). Complaints about palliative care were less common (n = 7, 3.4%), as well as about private medical specialists or institutions (n = 5, 2.4%), or private dental medicine providers (n = 5, 2.4%). Three complaints (1.4%) dealt with more than one category of healthcare institutions, and health institutions could not be identified in 3 complaints (1.4%). There was a total of 10 (4.8%) complaints against private healthcare institutions.

## Discussion

This is the first study, to the best of our knowledge, to map the complaints about violations of patients’ rights in Croatia according to the ECPR. The charter itself is not a law but has been developed as a consensus document and a valid tool for analysis and charting of patients’ complaints about violations of their rights [1].

The main finding of the study was that the official documentation about patients’ complaints was not informative with regard to the violation of their rights. Although there are several official pathways for patients to complain about violations of their rights, the official information was available only for less than half of the administrative and geographical units of the Republic of Croatia. The information on why these counties were omitted from the report was not available. It is highly unlikely that no patients submitted complaints in counties with missing data, as patients’ complaints received by non-governmental CAPR came from 20 out of 21 counties. A possible explanation for a greater number and wider geographical origin of complaints received by the CAPR is an overall low level of trust in institutions, especially in government and healthcare institutions [36, 37]. This is consistent with what has been found in study from the Netherlands, where the practice of bribery and corruption is still prevalent despite different healthcare system reforms conducted [38].

The analysis of the complaints to a specialized non-governmental organization for the protection of patients’ rights in Croatia showed that the most commonly reported violations could be mapped to the ECPR’s Right to Access, followed by the Right to Information and the Right to Safety. The majority of alleged violations were related to health care institutions in the City of Zagreb, followed by Split-Dalmatia County, the second-largest administrative unit in Croatia. This finding may be the reflection of the centralized healthcare system in Croatia, in which the large majority of official reference centres for the Ministry of Health and the largest tertiary health care centres are in Zagreb [39].

More than a half of all complaints dealt with hospitals (secondary level institutions), university hospital centres (tertiary level institutions), and specialists. We could not find studies from Croatia dealing with the comparison of patients’ complaints in primary and secondary healthcare settings, but our finding matches a recent systematic review of global literature, which showed that patients generally more often express concern regarding the secondary and tertiary level of care, including problems with communication and coordination [40]. Patients more often develop trusting and well-connected relations with their primary care physician [41], which has been proven to reduce the number of malpractice suits [42].

Patients’ rights outlined in the ECPR can be codified into five different groups in order to create a more workable analytical framework [43]. The first group is “Access to Healthcare”, including the Right to Preventative Measures, Right to Access, Right to Free Choice, Right to Respect Patients’ Time, Right to Innovation, and Right to Personalized Treatment. These rights are considered to be basic patient rights [44]. The Right of Access and the Right to Respect Patients’ Time were among the most commonly identified violations in our study, accounting for more than a third of all identified violations. Access to healthcare in Croatia is recognized as a problem, particularly for patients with lower socioeconomic status [45]. The Ministry of Health’s Strategic Plan for 2018–2021 emphasises the importance of equal healthcare access, especially for isolated parts and islands [39], but the is no data on whether the planned measures have been successful.

The Right to Health Innovations was identified in just a few cases. The knowledge of Croatian patients about clinical trials is overall rather low, and they are mostly not aware of trial registries and the availability of information for patients on clinical trials [46]. Furthermore, the number of clinical trials in Croatia is declining [47]. Patients’ complaints regarding the Right to Preventive Measures were also identified in just a few cases. This may be the reflection of Croatia’s long tradition of preventive public health [48]. The Croatian Institute for Public Health provides broad support for different public health campaigns, including vaccination and screening [49].

The other themes in the analytical framework of Mathuna et al. [43] are “Informed consent” (the Right to Information and Right to Consent), “Safety and Quality Assurance” (the Right to Observance of Quality Standards, the Right to Safety and the Right to Avoid Unnecessary Suffering and Pain), “Privacy and Confidentiality” (the Right to Privacy and Confidentiality) and “Redress” (the Right to Complain and the Right to Compensation). The right to information and right to informed consent is recognized in all Croatian laws regulating patients’ rights since 1993 [50]. It is particularly emphasized in Protection of Patients’ Rights Act, and patients have the right to information about their health, including the right to a second medical opinion, the right to information provided in an understandable way, as well as the right not to know [23]. The right to information was the second most often reported violation in our study, which may be related to the advancement of the Croatian health system from a paternalistic to the partner model [51]. Future studies are needed to assess this association.

In the Protection of Patients’ Rights Act [23], the right to shared decision-making includes both the right to be informed and the right to consent. A recent study demonstrated that the implementation of informed consent is satisfactory, but it identified problems in the informed consent process, such as low quality and comprehensibility of written forms used to obtain consent [52]. Moreover, shared decision-making education is not present in many medical schools’ curricula, and this aspect of the non-curriculum may be translated into everyday clinical practice [53]. On the other hand, there were only a few cases where we identified potential violations of the Right to Consent, which is most probably the consequence of strict legal rules, as providing treatment without consent is considered both criminal and civil offense in Croatia [23].

The violation of the right to complain was reported in a small number of cases. We believe this reflects the fact there are several instances where patients can file a complaint in Croatia (Fig. 1) [23, 30, 33, 39]. The Right to Compensation was identified in just a handful of cases. There are no data available on the number of lawsuits for compensation and possible trends, except for rare malpractice cases that gain media attention in Croatia, often following long and strenuous legal proceedings and huge monetary compensations [54].

In order to impact the quality of care, patients’ complaints should be comprehensive and context-specific [55–57]. While we categorized the complaints according to the rights outlined in the ECPR, the majority of complaints were actually questions about the healthcare system in Croatia. This contrasts similar research, where the majority of complaints have dealt with actual patients’ rights infringements, such as in Ireland [58]. Many questions about the healthcare system, as well as infringements of the Right to Information, which was the second most common patient complaint to the CAPR, could point out low health literacy in Croatia. Health literacy is a broad concept—a set of skills needed to function and understand the healthcare environment [59], ranging from understanding health information to health numeracy [60]. Low health literacy seems to be associated with poorer health outcomes [61], and different information about health or the healthcare system should be easily accessible to low-level health literacy patients [62]. Overall, health literacy in Croatia in hospitalized patients is less than adequate [63]. Future studies are needed to explore health literacy in the general public, as well as among those seeking help from the CARP, and identify specific characteristics and risk factors for low health literacy and how this may be linked to the understanding of patients’ rights.

Our study showed that the patient complaint system in Croatia is not well organized, as there are no developed coordinated procedures for responding to patient complaints, and no defined approaches to the legal protection, promotion, or recognition of patients’ rights, despite different official pathways to submit patients’ complaints. Whereas in other EU countries the relationship between health care professionals and patients is built on confidence and cooperation, gratified with person-centred communication, the Croatian health care system is still built on paternalistic doctor-patient relationships where patients often do not know the names of their specialist doctor [64].

Research into patient complaints is important as it helps identify problems in patient rights and safety. To achieve this, it is necessary to standardize how patient complaints are analysed and interpreted. Although patient complaints provide a unique insight into the problems that occur in the healthcare system, there is no systematic approach to evaluate and analyse these complaints at a central level in Croatia. The existence of several parallel pathways to report violations of patients’ rights in Croatia does not seem to increase the confidence of patients but is rather confusing as there are no clear instructions for patients about their rights and the procedures to protect them. The official bodies responsible for the protection of patients’ rights do not collaborate and do not follow protocols. Based on the results of our study, the recommendation for the Ministry of Health would be the adoption of a unique and clear pathway for complaints about health services, such as that of the National Health Service in the UK, which provides detailed instruction on how to complain to the health services, either online, in the waiting room or at the service provider website [65]. The patients filing the report can complain only to a single body, either directly to the NHS or the commissioner of services. In this way, health care organizations have to work together to ensure that the person filing the complaint receives an answer [66]. Future studies are needed to evaluate whether changes in healthcare influenced the number and the content of the patients’ complaints. Additionally, it would be interesting to see if potential interventions and CAPR activities with aim of improving patient healthcare literacy would influence the number and type of complaints.

Our study has also shown that there is a need for more educational efforts, both for the patients and healthcare workers [67]. For example, a simplified framework for teaching medical students about patient rights has been proposed [68].

### Limitations

As our study was cross-sectional in design, it is methodologically burdened by several limitations. The data from the Ministry of Health was incomplete and available only in an aggregate format, so we were not able to map the complaints of patients’ rights violations from this source to the ECPR. Eleven regional committees’ reports were missing. Secondly, we analysed patient complaints received via CAPR official email. This could be a potential selection bias as it could exclude older patients which do not commonly use electronic communication. Also, it is possible that someone filed several complaints, both to the Ministry and to CAPR, and that it was included in both datasets. However, as we were only able to analyse CAPR database for the ECPR because the data from the Ministry had been aggregated, we were not able to check for duplicates. Moreover, violations of patients’ rights could be reported by individuals with certain characteristics, who may not be representative of the whole population. The complaints made directly to the hospital or those including legal representatives without consulting CAPR or regional committees were not available for analysis. The dataset used in the study was from 2017 and 2018, as newer data was not available. Therefore, our analysis might not reflect the newest changes in the Croatian healthcare system, including the changes due to the COVID-19 pandemic. This study thus provides baseline evidence for future studies to follow changes in the number and type of patients’ complaints.

## Conclusions

The healthcare system in Croatia provides a complex framework for reporting and dealing with patients’ complaints, including both officially recognized bodies and non-government organizations, but this system is not effective in accurately capturing and reflecting the actual state of protection of patients’ rights in the Croatian health system. Publicly available data from regional committees do not specify complaints related to possible violations of patients’ rights, and the comments sent to non-governmental patients’ rights organizations are not official and are probably not fully representative. More transparency and a clear process of lodging complaints are needed in order to better understand patients’ needs, resolve allegations, prevent future complaints, thus increasing the quality and safety of the health care system.

## Data Availability

The datasets generated and/or analysed during the current study are not publicly available due to privacy but are available from the corresponding author on reasonable request.

## Abbreviations

ECPR: European Charter of Patients’ Rights
HCA: Healthcare Act
PRPA: Protection of Patients’ Rights Act
CAPR: Croatian Association for the Protection of Patient Rights
